# Supplementary material for: Lightwave-driven scanning tunnelling spectroscopy of atomically precise graphene nanoribbons
Source: Nat Commun. 2021 Nov 23;12:6794. doi: 10.1038/s41467-021-26656-3 (PMC8611099; doi:10.1038/s41467-021-26656-3)
Supplement: Supplementary file 1 — Supplementary Information [file 41467_2021_26656_MOESM1_ESM.pdf]

Supplementary Information for

Lightwave-driven scanning tunnelling spectroscopy of atomically precise graphene nanoribbons

S. E. Ammerman[1,†], V. Jelic[1,†], Y. Wei[1], V. N. Breslin[1], M. Hassan[1], N. Everett[1], S. Lee[1], Q. Sun[2,‡], C. A. Pignedoli[2], P. Ruffieux[2], R. Fasel[2,3] and T. L. Cocker[1]*

[1]Department of Physics and Astronomy, Michigan State University, East Lansing, MI 48824, USA

[2]Empa, Swiss Federal Laboratories for Materials Science and Technology, 8600 Dübendorf, Switzerland

[3]Department of Chemistry, Biochemistry and Pharmaceutical Sciences, University of Bern, 3012 Bern, Switzerland

*Correspondence to: cockerty@msu.edu

[†]These authors contributed equally to this work

[‡]Present address: Materials Genome Institute, Shanghai University, 200444 Shanghai, China

This file includes:

Supplementary Figures 1 to 9

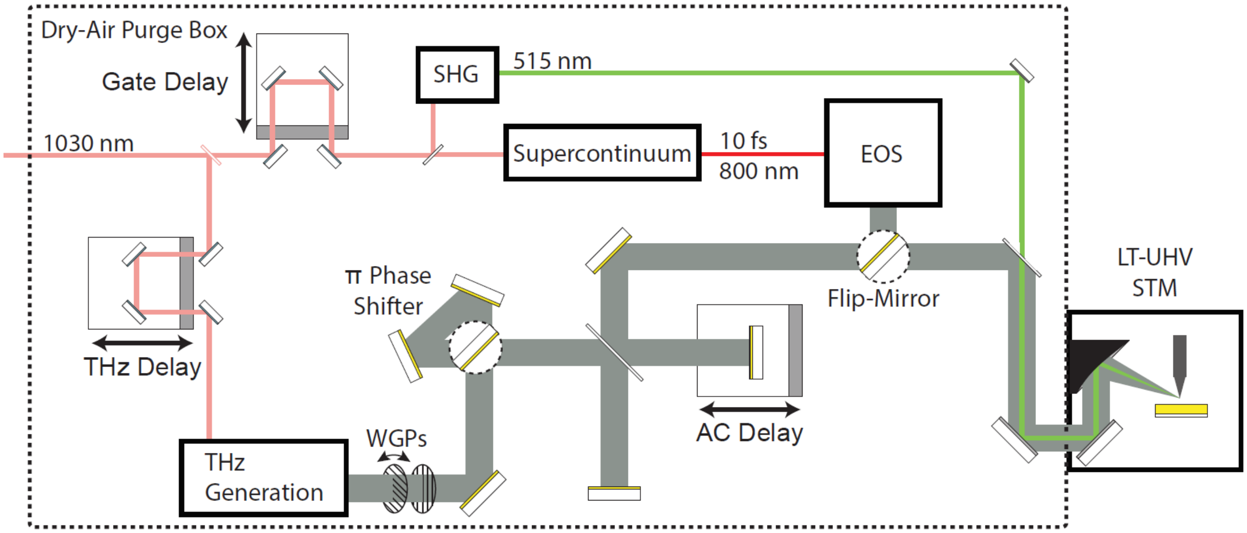

Supplementary Figure 1 | Optical Setup for THz-STM of 7-AGNRs and THz waveform characterization. SHG – second harmonic generation; EOS – electro-optic sampling; AC – autocorrelator; LT-UHV STM – low-temperature ultrahigh-vacuum scanning tunnelling microscope; WGPs – wire-grid polarizers (10 μm wire with 30 μm spacing).

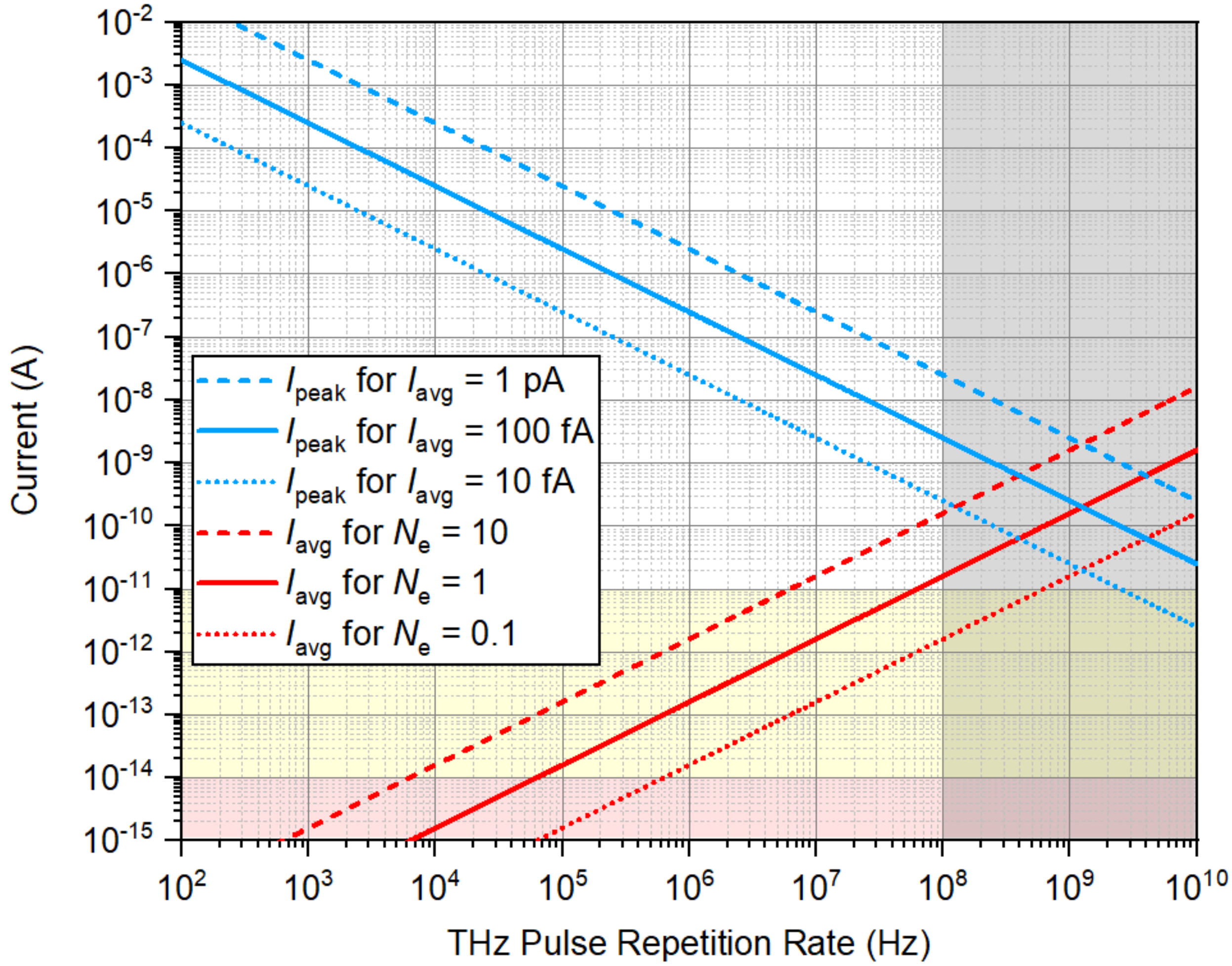

Supplementary Figure 2 | Comparison of time-averaged and peak-transient THz-pulse-driven tunnel currents at various repetition rates. In red, the average THz-pulse-driven tunnel current (I_{avg}) is shown for a constant number of rectified elementary charges per THz pulse: N_e = 10 (dashed red line), N_e = 1 (solid red line) and N_e = 0.1 (dotted red line). In blue, the peak instantaneous THz-pulse-driven tunnel current (I_{peak}) is shown for a constant average THz-pulse-driven tunnel current: I_{avg} = 1 pA (dashed blue line), I_{avg} = 100 fA (solid blue line) and I_{avg} = 10 fA (dotted blue line). The blue curves assume that the THz pulse voltage is active for 400 fs (an estimation based on past measurements[1,2]). The shaded box in red shows the typical tunnel current detection limit of conventional STM systems that utilize lock-in detection (~10 fA), while the shaded box in yellow shows the typical range of the tunnel current for a high gain preamp operating in constant-current mode (active feedback loop). The dark shaded box on the right highlights an unexplored region that requires new technological advancements to achieve the necessary THz electric fields that can drive tunneling across the junction using multi-GHz repetition rate laser systems.

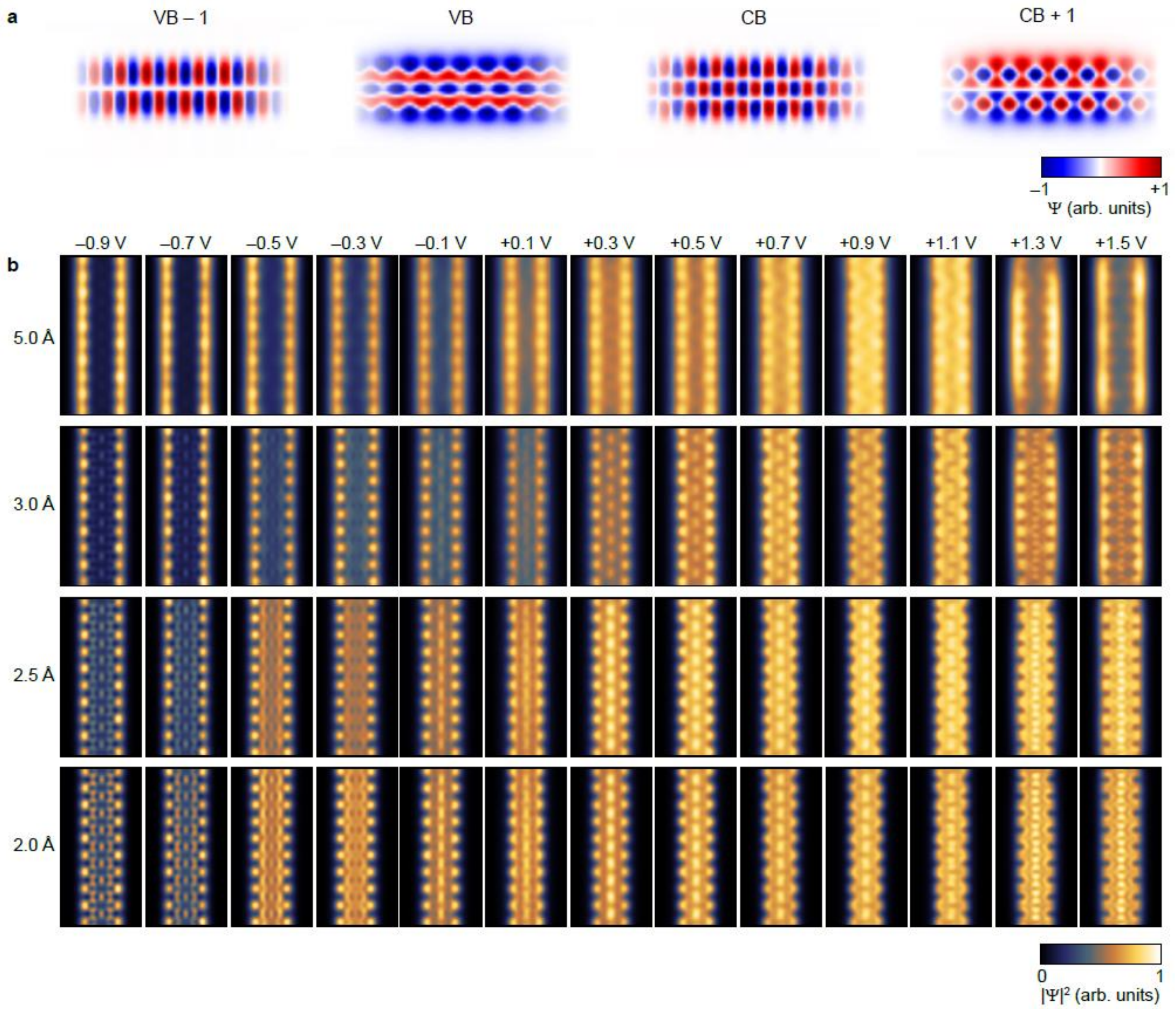

Supplementary Figure 3 | Simulated planar cross-sections of the electron wavefunction and probability density for a 7-AGNR as a function of distance and energy. **a**, Spatial distribution of the electron wavefunction, Ψ, for energies near the second valence band (VB–1), valence band (VB), conduction band (CB) and second conduction band (CB+1) calculated using density functional theory at a tip–sample distance of z = 2.0 Å. **b**, Spatial distribution of the electron probability density, $|\Psi|^2$, at energies ranging from 0.9 eV below to 1.5 eV above the Fermi level (see Methods). Image size 2 nm × 4 nm. The height of the planar cross-section is referenced to the atomic lattice of the 7-AGNR. The colormap was set independently for each image. The calculation in **a** was performed with the 7-AGNR in the gas phase, while the calculation in **b** had the 7-AGNR adsorbed on an Au(111) surface.

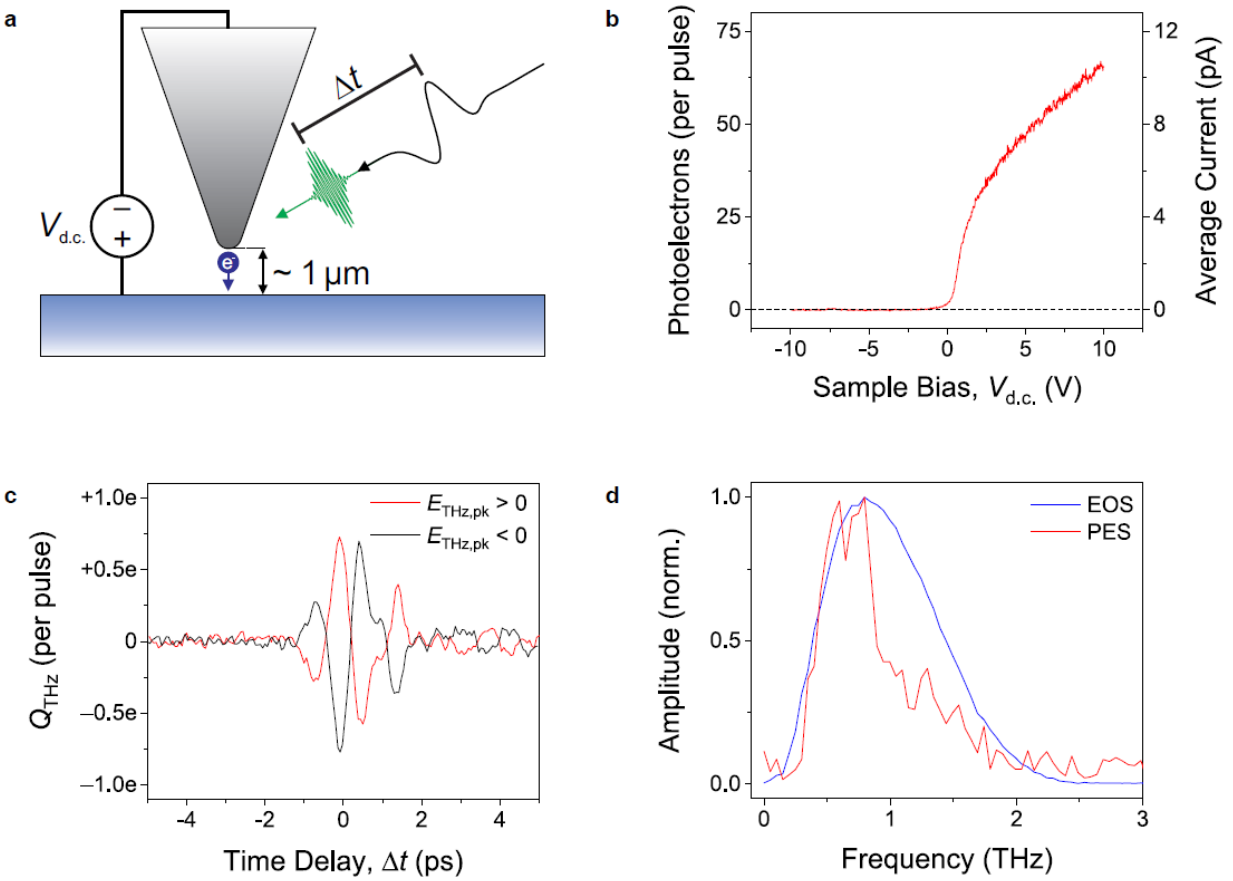

Supplementary Figure 4 | Measuring the THz waveform at the tip apex using photoemission sampling (PES). a, Schematic diagram of the experimental setup for ultrafast PES. **b**, Current–voltage relationship for ultrafast photoemission using an excitation pulse-train with a repetition rate of 1 MHz, a center wavelength of 515 nm, a pulse duration of < 230 fs and a pulse energy of 30 nJ. **c**, Measured THz waveforms at the tip apex acquired for $E_{THz,pk}$ = +180 V/cm (red curve) and $E_{THz,pk}$ = –180 V/cm (black curve) incident field. **d**, Amplitude spectrum for the EOS waveform in Fig. 2e (blue curve) and PES waveform in **c** (red curve).

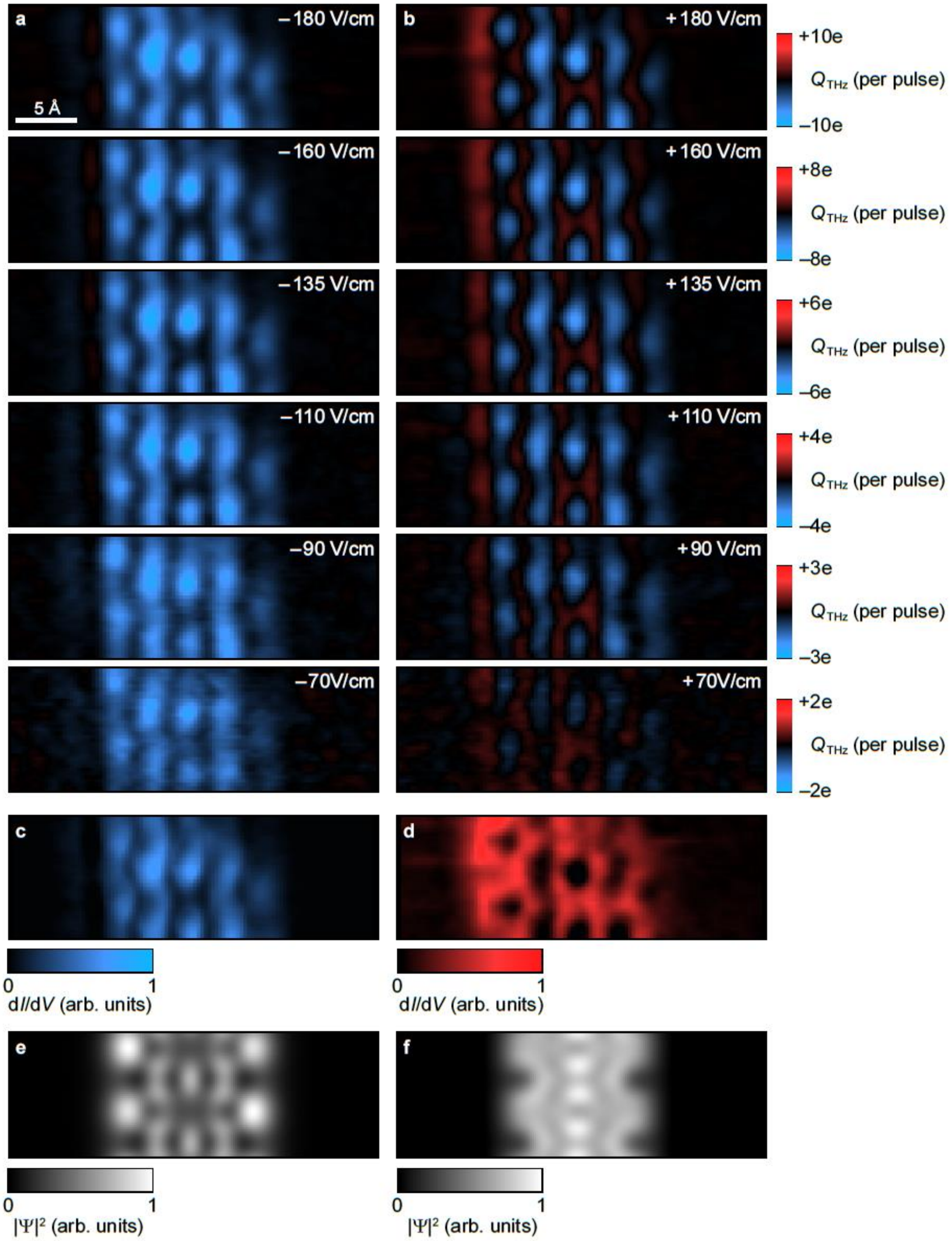

Supplementary Figure 5 | THz-STM field dependent imaging and extracted THz-STS d*I*/d*V* maps. a,**b**, THz-STM constant-height rectified charge maps acquired at several $E_{THz,pk}$ using a negative-field-dominant THz pulse (**a**) and a positive-field-dominant THz pulse (**b**). The images were acquired with the tip at $z = z_0 - 4$ Å. A 5×5 smoothing filter was applied to the dataset. **c**,**d**, The valence band THz-STS image (**c**) and conduction band THz-STS image (**d**) were extracted from the dataset in **a** and **b** by generating a 12-point Q_{THz}-$E_{THz,pk}$ curve at every pixel of the image and then applying the model outlined in Methods. **e**,**f**, Simulated electron probability calculated using DFT at a tip height of 2 Å with $V = -0.9$ V (**e**) and $V = +1.3$ V (**f**).

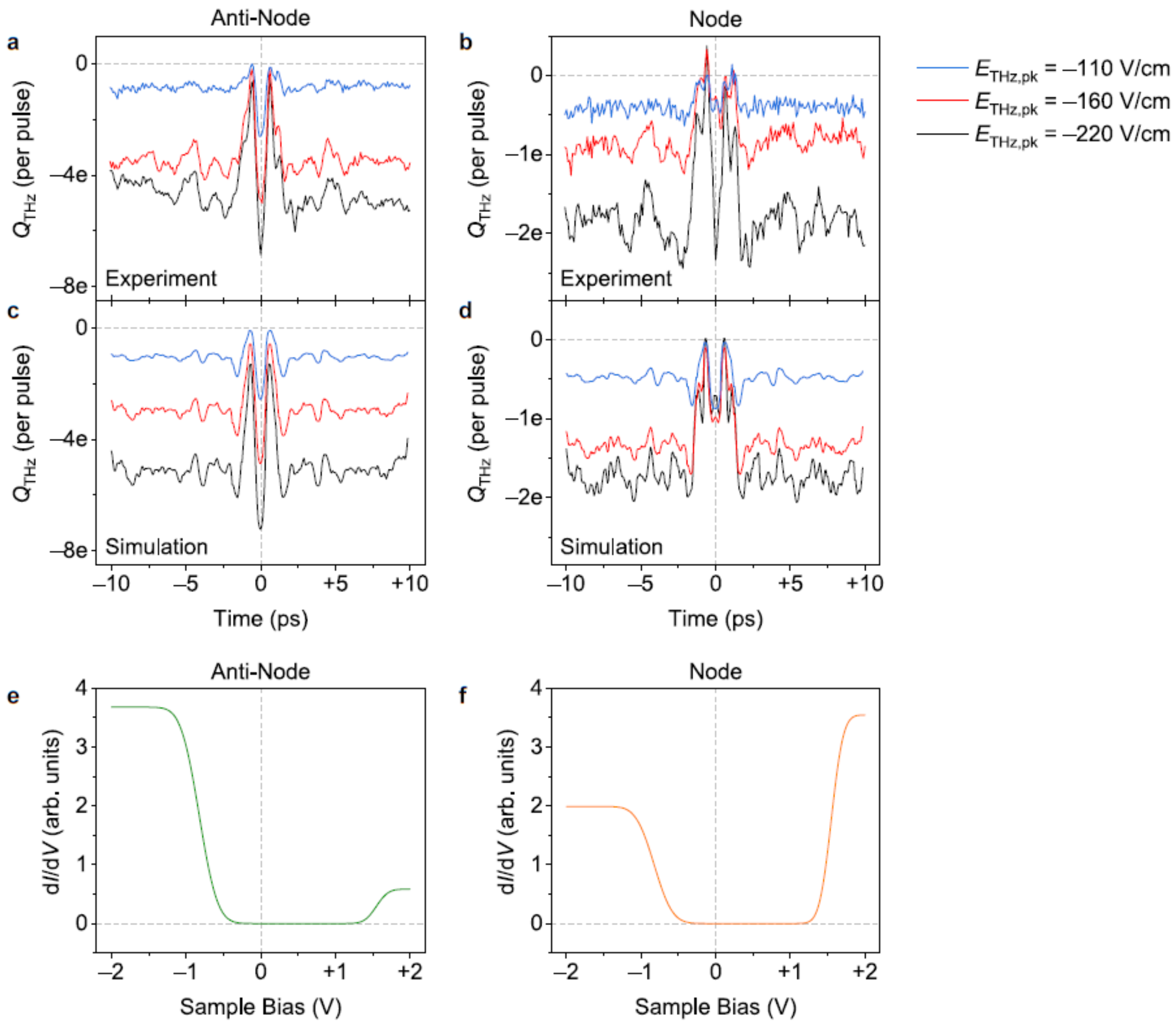

Supplementary Figure 6 | THz-STM autocorrelations of a 7-AGNR on Au(111). **a**,**b**, Experimental THz pulse autocorrelations acquired at zero d.c. bias ($V_{d.c.}$ = 0 V) and at constant height ($z = z_0 - 4$ Å) with the tip positioned at the anti-node (**a**) and node (**b**). **c**,**d**, Simulated THz pulse autocorrelations for the the anti-node (**c**) and node (**d**) tip positions using the PES waveform in Fig. 2e. The nonlinearity of the *I*–*V* curve that rectifies the THz pulse within the model was determined from the t = 0 ps and t = 10 ps values of the experimental autocorrelations. **e**,**f**, Model d*I*/d*V* for the anti-node (**e**) and node (**f**) tip positions.

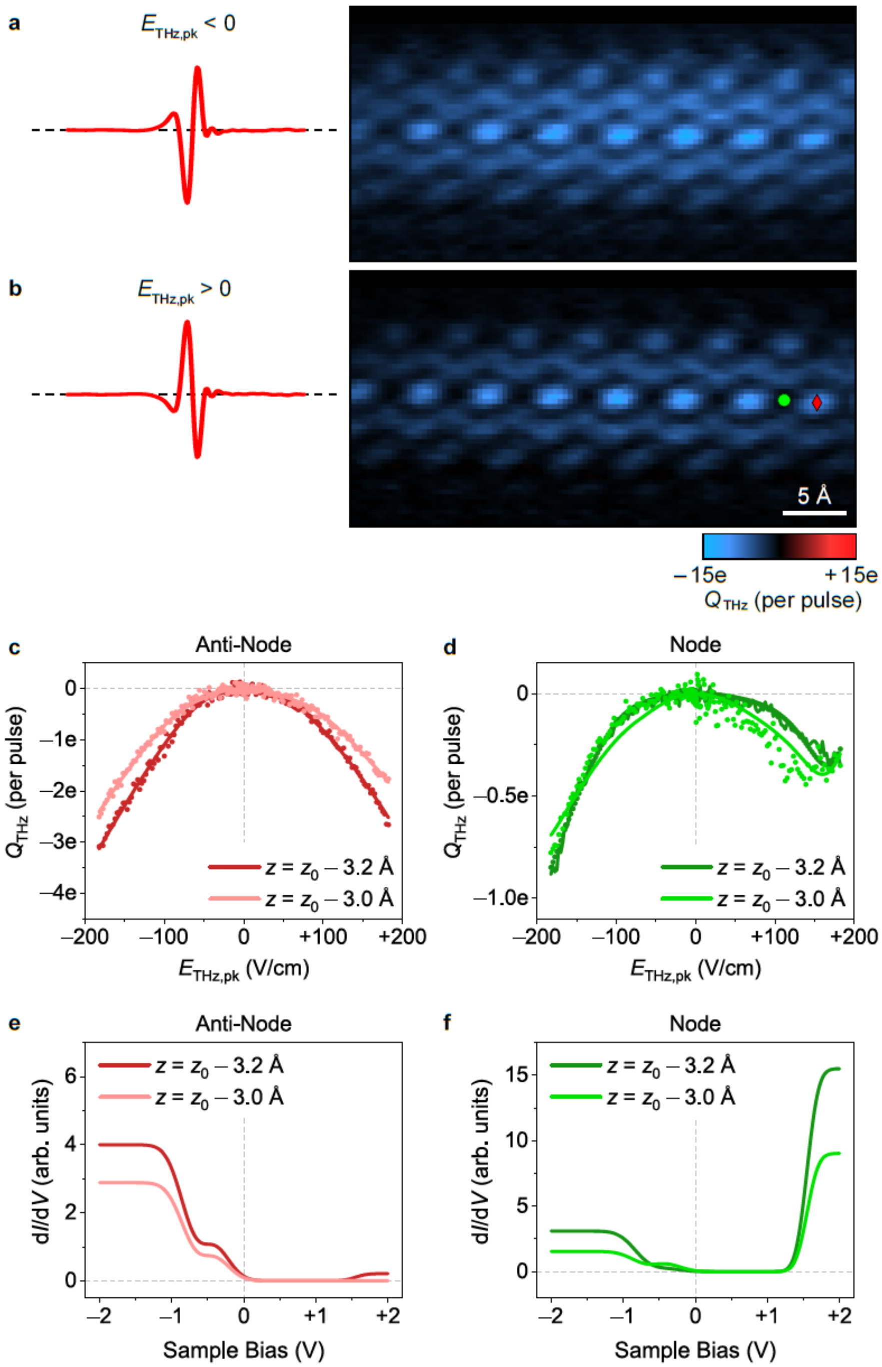

Supplementary Figure 7 | THz-STM imaging and spectroscopy of another 7-AGNR with a different tip apex. **a**,**b**, THz-STM constant-height rectified charge maps acquired at $E_{\mathrm{THz,pk}}$ = –135 V/cm (**a**) and $E_{\mathrm{THz,pk}}$ = +135 V/cm (**b**), with $V_{\mathrm{d.c.}}$ = 0 V and $z = z_0$ – 3.0 Å for both images. The red triangle and green circle indicate the THz-STS locations for the anti-node and node, respectively. **c**,**d**, Measured (circles) and simulated (solid lines) Q_{THz}–$E_{\mathrm{THz,pk}}$ curves at $z = z_0$ – 3.2 Å (dark lines/symbols) and $z = z_0$ – 3.0 Å (light lines/symbols) with the tip positioned at the anti-node (**c**) and node (**d**) of the valence band LDOS ($V_{\mathrm{d.c.}}$ = 0 V). **e**,**f**, Model dI/dV extracted from the fits in **c** and **d** for the anti-node (**e**) and node (**f**) tip positions, respectively.

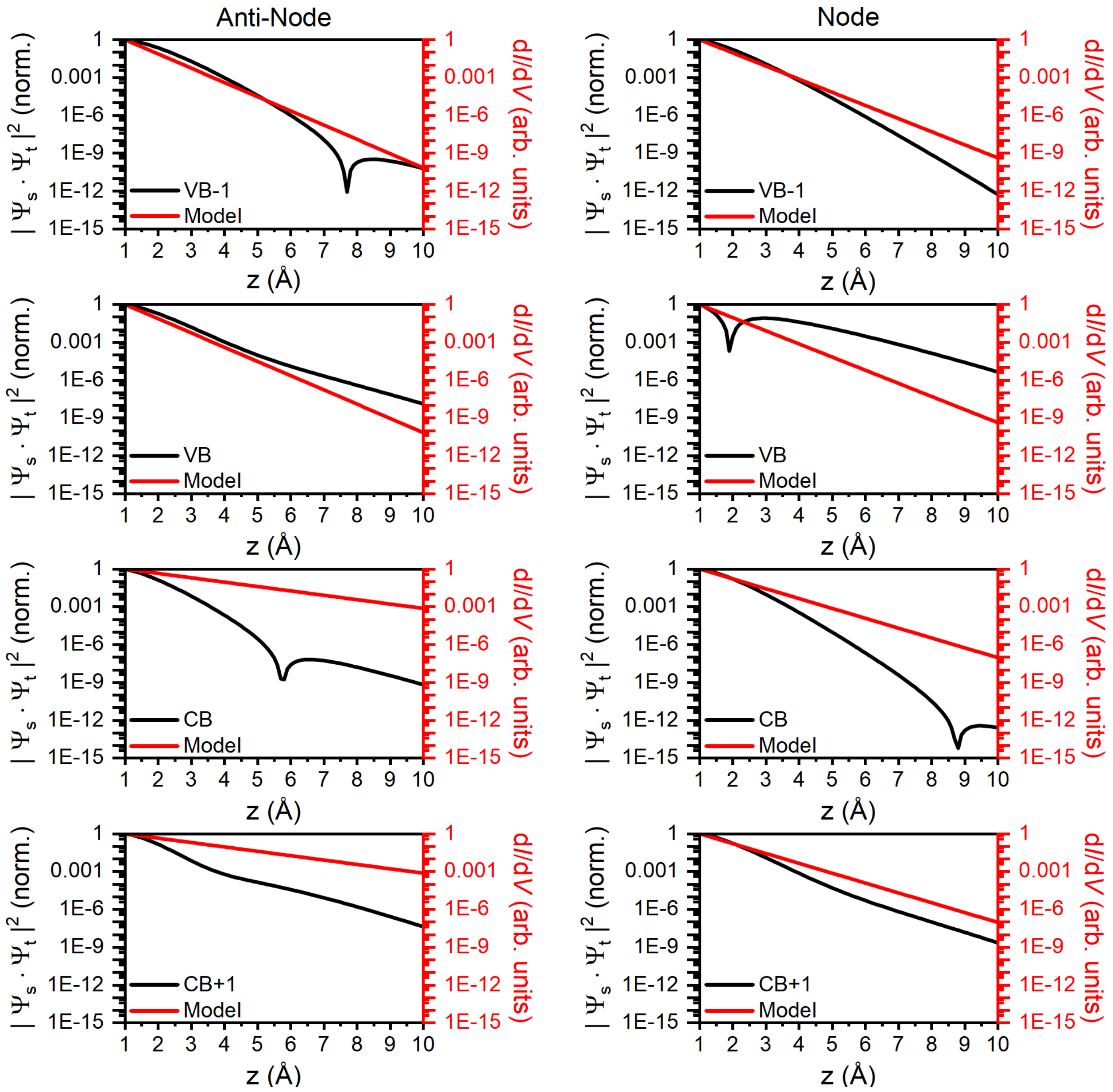

Supplementary Figure 8 | Comparison of the theoretical and experimental vertical decays at the anti-node and node positions. The theoretical vertical decay (solid black line) was calculated using a tight-binding model for the sample wavefunction (Ψ_s) that was convoluted with an *s*-wave tip (Ψ_t) following the Tersoff-Hamann approximation[3]. The theoretical decays (solid black line) are shown alongside vertical cuts through the extracted d*I*/d*V* of Fig. 4c and 4d (solid red line). The theoretical decays and extracted d*I*/d*V* are artificially normalized to the extracted d*I*/d*V* at z = 0 Å for easier comparison. The VB and VB-1 decays are compared to d*I*/d*V* at $V = -0.9$ V while the CB and CB+1 decays are compared to d*I*/d*V* at $V = +1.3$ V.

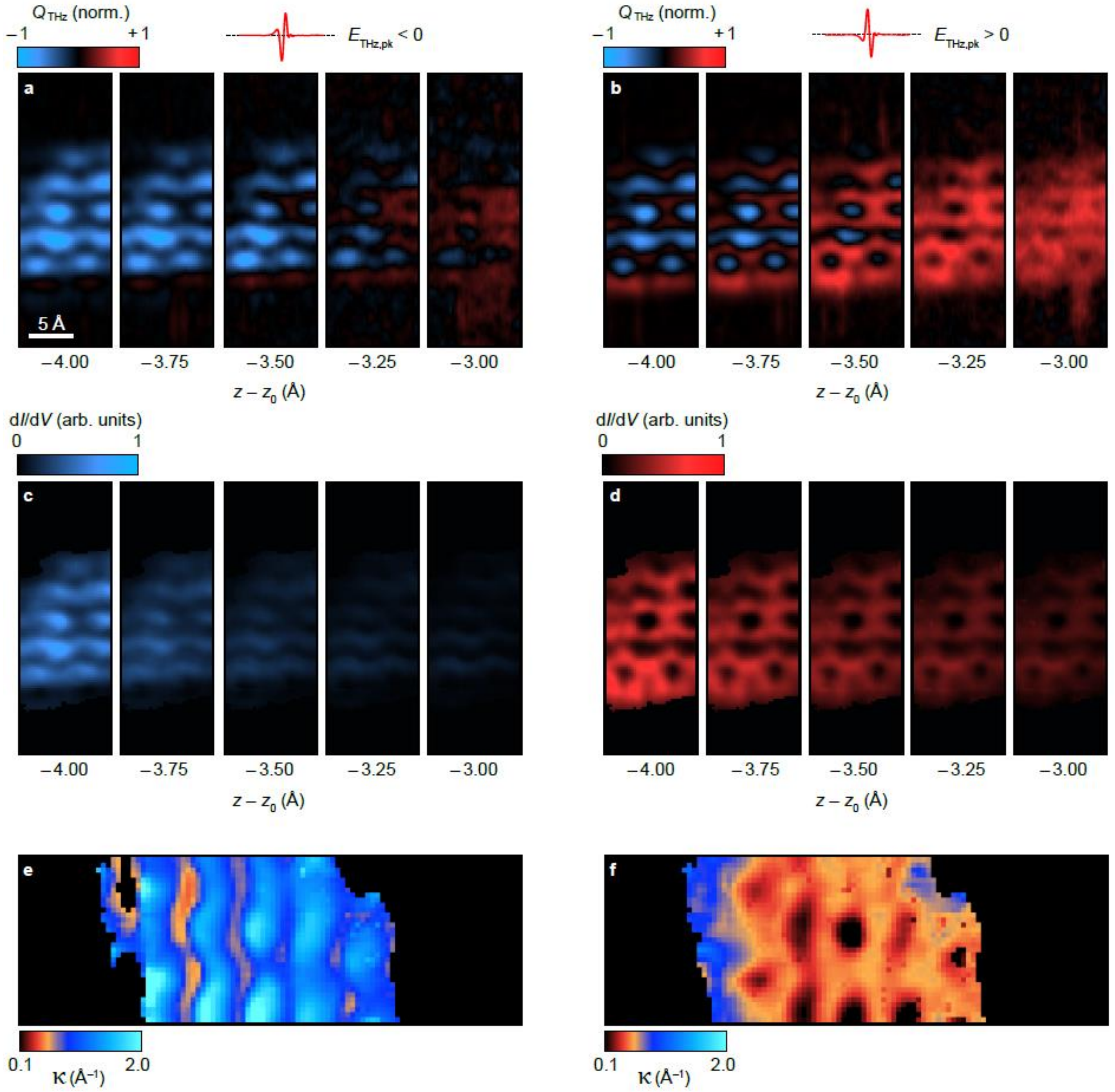

Supplementary Figure 9 | THz-STM height dependent imaging and tomographic d*I*/d*V* reconstruction. a,**b**, THz-STM constant-height rectified charge maps acquired at multiple tip–sample distances with $E_{\mathrm{THz,pk}} < 0$ (**a**) and $E_{\mathrm{THz,pk}} > 0$ (**b**). A 5×5 smoothing filter was applied to the dataset in **a** and **b**. **c**,**d**, Vertical decay of the differential conductance for occupied (**c**) and unoccupied (**d**) states. **e**,**f**, Map of the inverse decay constant, κ, extracted from the dataset in **a** and **b** by creating a Q_{THz}-z curve at each pixel and applying the model outlined in Methods for a constant $E_{\mathrm{THz,pk}}$. The regions within **e** and **f** that are represented by zero on the colormap (black) are artificially inserted because the local value for Q_{THz} and/or the d*I*/d*V* amplitude at the corresponding bias is negligible and κ cannot be determined.

References

1. Cocker, T. L. *et al.* An ultrafast terahertz scanning tunnelling microscope. *Nat. Photonics* **7**, 620–625 (2013).
2. Jelic, V. *et al.* Ultrafast terahertz control of extreme tunnel currents through single atoms on a silicon surface. *Nat. Phys.* **13**, 591–598 (2017).
3. Tersoff, J & Hamann, R. Theory and Application for the Scanning Tunneling Microscope. *Phys. Rev. Lett.* **50**, 1998–2001 (1983).
